# Radiomics‐Based Prognostication in Primary Sclerosing Cholangitis: A Proof‐of‐Concept Study

**DOI:** 10.1111/liv.70348

**Published:** 2025-09-27

**Authors:** Laura Cristoferi, Cesare Maino, Davide Paolo Bernasconi, Ilaria Ripamonti, Miki Scaravaglio, Alberto Savino, Eugenia Pesatori, Alessio Gerussi, Eugenia Nofit, Olga Falco, Daphne D'Amato, Francesca Gallivanone, Chiara Alberzoni, Raffaella Viganò, Chiara Mazzarelli, Luca Saverio Belli, Marco Emilio Dinelli, Massimiliano Mutignani, Maria Grazia Valsecchi, Rocco Corso, Pietro Invernizzi, Marco Carbone, Davide Ippolito, Elisabetta De Bernardi

**Affiliations:** ^1^ Division of Gastroenterology, Center for Autoimmune Liver Diseases European Reference Network on Hepatological Diseases (ERN RARE‐LIVER), Fondazione IRCCS San Gerardo dei Tintori Monza Italy; ^2^ Department of Diagnostic Radiology Fondazione IRCCS San Gerardo dei Tintori Monza MB Italy; ^3^ Bicocca Bioinformatics Biostatistics and Bioimaging Centre‐B4, School of Medicine and Surgery University of Milan‐Bicocca Monza MB Italy; ^4^ ASST Grande Ospedale Metropolitano Niguarda Clinical Research and Innovation Unit Milan Italy; ^5^ Department of Medicine and Surgery University of Milano‐Bicocca Monza Italy; ^6^ Department of Gastroenterology ASST Papa Giovanni XXIII Bergamo Italy; ^7^ Institute of Molecular Bioimaging and Physiology, National Research Council (IBFM‐CNR) Milan Italy; ^8^ Hepatology and Gastroenterology Unit ASST Grande Ospedale Metropolitano Niguarda Milan Italy; ^9^ Interventional Endoscopy Foundation IRCCS San Gerardo dei Tintori Monza Italy; ^10^ Digestive Endoscopy Unit ASST Grande Ospedale Metropolitano Niguarda Milan Italy

**Keywords:** artificial intelligence, autoimmune liver diseases, quantitative radiology, radiomics, risk stratification, surrogate biomarkers

## Abstract

**Background and Aim:**

Risk assessment in primary sclerosing cholangitis (PSC) by magnetic resonance imaging (MRI) relies on semi‐quantitative analysis, which can result in interpretation variability. Radiomics may offer a quantitative approach for risk stratification. This study aims to explore and validate MRI‐derived radiomic features to identify high‐risk PSC patients.

**Methods:**

In this prospective study (January 2019–December 2022), consecutive PSC patients undergoing routine gadoxetate disodium‐enhanced MRI were recruited. Using PyRadiomics, whole liver parenchyma features were extracted from five MRI sequences according to the Image Biomarker Standardisation Initiative (IBSI). Patients were categorised into risk groups based on the Mayo risk score (MRS) and liver stiffness measurement (LSM). Features associated with high‐risk patients were selected and validated in an independent cohort. A survival analysis was conducted in the combined cohort to assess the prognostic value of the radiomic features for clinical events.

**Results:**

One hundred and two PSC patients were enrolled in this study. Five radiomics features were associated with high risk in the training cohort. In the validation setting, *GLRLM‐Run Entropy* in the fat‐saturation T2 weighted imaging (FS‐T2W) sequence was the only significant feature, with an odds ratio of 3.90 (CI 1.46–10.42, *p* = 0.007) for MRS and 2.97 (CI 1.33–6.66, *p* = 0.008) for LSM. Its prognostic potential on clinical outcome was confirmed by Cox regression analysis in the combined cohort (hazard ratio per 0.1 increase = 1.480, CI 1.226–1.786), showing excellent predictive performance (C‐index = 0.857).

**Conclusions:**

*GLRLM‐Run Entropy* in FS‐T2W is a novel radiomics‐based biomarker for risk stratification in PSC patients. It is quantitative, standardised, easy to compute and cost‐free, positioning it as a potential key innovation in PSC radiology‐based biomarkers.

**Trial Registration:**

Clinicaltrial.gov ID: NC705618145

**Abbreviations**
AI: artificial intelligence
ALP: alkaline phosphatase
AOS: Amsterdam Oxford Score
AST: aspartate aminotransferase
AUC: area under the curve
DWI: diffusion‐weighted imaging
EASL: European Association for the Study of the Liver
FS: fat‐saturation
GLRLM: Gray Level Run Length Matrix
GRE: gradient echo
HBP: hepatobiliary phase
HR: hazard ratio
IBD: inflammatory bowel disease
IBSI: image biomarker standardisation initiative
IQR: interquartile range
LASSO: least absolute shrinkage and selection operator
LSM: liver stiffness measurement
LT: liver transplantation
MC: medical centre
MELD: model for end‐stage liver disease
ML: Machine Learning
MR: magnetic resonance
MRCP: magnetic resonance cholangiopancreatography
MRI: magnetic resonance imaging
MRS: Mayo Risk Score
OR: odds ratio
PSC: primary sclerosing cholangitis
ROI: region of interest
STAPLE: simultaneous truth and performance level estimation
TDA: topological data analysis
TR: repetition time
TSE: turbo spin echo
ULN: upper limit of normal

**Summary**

- In this study, radiomics have been applied to standardised MRI images to identify and validate biomarkers that can predict risk progression in primary sclerosing cholangitis (PSC).
- A radiomics feature has been pinpointed, *GLRLM‐Run Entropy* in FS‐T2W, as a key predictor of high‐risk patients, offering a novel, quantitative approach to enhance the precision of PSC risk stratification and potentially guide a personalised patient management.

## 1 Introduction

The absence of validated biomarkers in clinical practice in primary sclerosing cholangitis (PSC) poses challenges in discriminating patients at higher risk of clinical events, designing clinical trials and testing novel drugs towards surrogate endpoints. In addition, the scarcity of symptoms in early disease stages limits timely interventions. Although several risk assessments and biomarkers have been proposed to monitor disease progression, their effectiveness is limited as they rely on biochemical indicators that may not consistently reflect changes in parenchymal and ductal structures [1].

Magnetic resonance imaging (MRI) with cholangiopancreatographic sequences (MRCP) is the gold standard for the diagnosis, staging and monitoring of PSC progression and its complications over time [1]. In recent years, substantial efforts have focused on developing imaging‐based risk prediction models. Nevertheless, the clinical application of these models is limited by their reliance on qualitative or semi‐qualitative variables resulting in suboptimal performance, limited reproducibility and poor generalizability [2].

Radiomics offers a potential solution by extracting quantitative information from MRI, identifying patterns not detectable by the human eye [3]. To date, radiomics and artificial intelligence (AI) in autoimmune liver disease have been poorly explored [4].

Its application led to the creation of image processing software MRCP+ software (Perspectum Ltd., UK) for biliary metrics from MRCP 3D sequences, showing a promising link to clinical outcomes in several studies [5, 6, 7, 8]. However, it focuses on MRCP sequences, neglecting the evaluation of liver parenchyma around inflamed bile ducts. This might be a key limitation in PSC where the evaluation of liver parenchyma can vary significantly among operators [2].

The analysis of the liver using radiomics and an AI‐based approach using algebraic topology machine learning on contrast‐enhanced T1‐weighted MR images of PSC patients has shown good results in predicting short‐term hepatic decompensation [9]. However, its technical complexity makes implementation difficult in centres lacking bioinformatics staff, and its reproducibility is still uncertain. Our study aimed to identify and validate radiomics features extracted on different MRI sequences and compliant with the Image Biomarker Standardisation Initiative (IBSI) [10] using a free and globally available tool, able to classify PSC patients at higher risk of disease progression.

## 2 Methods

### 2.1 Patients' Selection

From January 2020 to October 2021, all consecutive patients with a diagnosis of PSC who underwent gadoxetate disodium–enhanced (Gd‐EOB‐DTPA) MRI using a standardised protocol of acquisition at *Fondazione IRCCS San Gerardo dei Tintori* were included in the study. Diagnosis of PSC was established according to European guidelines [11].

Inclusion criteria for this study were as follows: age ≥ 16 years, liver function tests (LFTs) obtained within 2 weeks before the MRI, and normal kidney function (estimated glomerular filtration rate > 60 mL/min/1.73 m^2^).

Exclusion criteria were as follows: small duct PSC, known hepatic comorbidities (i.e., viral hepatitis, autoimmune hepatitis, current or past viral hepatitis, metabolic dysfunction‐associated steatohepatitis (MASH)), previous surgery on the biliary tree or liver transplantation (LT), hepatic or biliary neoplasms and decompensated cirrhosis before or at the time of MRI.

Patients were allocated to either a training cohort consisting of 56 patients or a validation cohort consisting of 43 patients, using temporal criteria: patients with MRI performed in 2020 were included in the training cohort, and patients with MRI performed in 2021 were included in the validation cohort.

The study was conducted in accordance with the guidelines of the Declaration of Helsinki and the principles of good clinical practice. All participants provided written informed consent. The study was approved by the University of Milan‐Bicocca Research Ethics Committee (study name: PSC Database).

### 2.2 Clinical Data

The authors conducted a review of medical records to gather clinical data. Specifically, LFTs (aspartate aminotransferase [AST], alanine aminotransferase [ALT], alkaline phosphatase [ALP], total serum bilirubin, serum albumin), serum creatinine, estimated glomerular filtration rate and platelets, closest to the date of MRI, were selected for analysis. Mayo risk score (MRS) was calculated [12]. Amsterdam Oxford Score (AOS), ANALI score with and without gadolinium (GD) [13] and model for end‐stage liver disease (MELD) have been calculated for all the patients enrolled [14, 15, 16]. Liver stiffness measurement (LSM) was performed within 3 months before or after MRI by trained operators. Only measurements with 10 valid readings and interquartile range (IQR)/median ratio < 0.3 were considered reliable.

### 2.3 Risk Classification

Surrogate predictive markers of disease progression were used as the reference in this study. Patients with PSC were classified into high‐risk or low‐risk groups as described below.

- MRS: patients were categorised into (1) low risk, ≤ 0; (2) intermediate risk, > 0–2; and (3) high risk, > 2 [12, 17]. For the analysis, the intermediate‐ and high‐risk categories were grouped together as in other studies of similar size [17]; we defined MRS > 0 as high risk to avoid small numbers in a group.
- LSM: high‐risk was defined as LSM > 9.6 kPa as this cut off is associated with histologically assessed fibrosis [18]. Furthermore, preliminary data from a prospective study with the collaboration of the International PSC study group (IPSCsg) confirmed the cut off of 9.6 kPa to discriminate patients at higher risk of clinical event development [19].

### 2.4 MRI Technique

All the MRI exams were performed using a 1.5 Tesla MRI scanner (Ingenia, Philips). The choice of the sequences to include for the analysis of the liver parenchyma has been made following the recommendation of the IPSCsg [20] and according to their ability to identify signs of fibrosis and inflammation in the liver parenchyma surrounding the biliary tract [21].

Sequences selected for the analysis are reported in Table S1 with the acquisition parameters. Only studies with all the MR images free of significant motion artefacts were selected by an expert biliary radiologist and included in the analysis.

### 2.5 Liver Segmentation, Feature Extraction and Selection

Methodology description regarding semi‐automated liver segmentation, radiomics region of interest (ROI) definitions, radiomic feature extraction and selection is detailed in the Methods, Sections 1–3: Appendix S1. This includes procedures for image preprocessing, liver segmentation, feature extraction with PyRadiomics and the definition of each class (Table S2) and the strategies applied for feature selection. Additionally, the portability of radiomics features over simpler liver segmentations was assessed and is also discussed in the Methods, Section 1: Appendix S1.

### 2.6 Statistical Analysis

To account for interlaboratory variability, ALP, ALT, AST and total bilirubin are expressed as a multiple of their respective upper limit of normal values (ULNs). Categorical data are presented as numbers (percentages), while continuous variables are expressed as medians, interquartile ranges (Q1–Q3). The distribution of the variables was compared between training and validation cohorts using chi‐squared test for categorical variables and Wilcoxon rank‐sum test for continuous variables.

The association of selected radiomics features with high‐risk classification (Mayo risk score > 0 and LSM > 9.6 kPa) was estimated using odds ratios (OR) from logistic regression, while the discriminative ability to identify patients at higher clinical risk was assessed using area under the curve (AUC).

To determine the performance of the selected radiomic features to predict clinical events in time, the training and validation cohorts were combined into a merged cohort to increase the number of observed clinical events.

The clinical outcomes considered were the first occurrence of: liver transplantation, hepatic decompensation and liver‐related death. The follow‐up time is intended as the time from MRI performance and the first occurrence of a clinical event or last clinical follow‐up.

We performed univariate analysis of prognostic variables using Cox's proportional hazards regression. The relatively small number of clinical events observed did not allow us to perform a multivariable regression analysis with selected features. Thus, to assess the prognostic value of the selected radiomic features adjusted for other known clinical factors, we performed several bivariable Cox models including the radiomic features alongside with established prognostic biomarkers in PSC. We also computed the C‐index of the univariable and bivariable models to assess the discriminatory ability to identify patients at higher risk of clinical events. The difference in the C‐index between a univariable model with a single the selected radiomic feature allows us to assess the predictive power added by the radiomic feature. Statistical analyses were performed using R software (version 4.3.2).

## 3 Results

### 3.1 Patients' Characteristics

One hundred and two consecutive contrast‐enhanced MRIs of patients with large duct PSC acquired with a standardised acquisition protocol were enrolled in the study period (January 2020–October 2021). Three MRIs have been excluded by the analysis due to the presence of important respiratory artefacts on at least one of the analysed MR sequences. The overall cohort included 99 patients: the first 56 patients were allocated to the training cohort and the next 43 patients into the validation one, using temporal criteria. The baseline characteristics of patients in the training and validation cohorts are shown in Table 1; no significant differences were found between the cohorts. Consistently with other recent series, median age at MRI was 35 years (interquartile range [IQR] 27–55) and 45 years (IQR 34–57); 50.0% and 37.2% were female, and 74.1% and 62.8% had an IBD in the training and validation cohorts, respectively. Patients considered at high risk according to MRS (> 0) and LSM (> 9.6 kPa) were 12 (22.2%) and 6 (14.0%), and 14 (26.4%) and 13 (31.7%), in the training and validation cohorts, respectively.

**TABLE 1:** Training and validation cohort patients' characteristics.

Demographics and clinical variables	Training cohort	Validation cohort	*p*
	*N* = 56	*N* = 43	
	Median or *N* [Q1; Q3 or %]	Median or *N* [Q1; Q3 or %]	
Age at MRI (years)	35 [28; 55]	45 [34; 57]	0.062
Time from diagnosis to MRI (months)	44.9 [12.1–83.3]	86.1 [42.9–113.2]	0.0515
Female gender	28 [50.0]	16 [37.2]	0.292
IBD	40 [74.1]	27 [62.8]	0.330
Cirrhosis	7 [13.0]	2 [4.7]	0.294
Previous GE variceal bleeding	2 [3.7]	0 [0.0]	0.578
Total bilirubin	0.75 [0.50; 1.31]	0.60 [0.50; 1.51]	0.610
ALP × ULN	1.04 [0.69; 2.08]	0.95 [0.61; 1.93]	0.495
AST × ULN	0.75 [0.56; 1.25]	0.82 [0.53; 1.27]	0.468
ALT × ULN	0.89 [0.52; 1.41]	0.72 [0.42; 1.46]	0.269
LSM (kPa)	6.60 [4.95; 10.20]	6.10 [4.60; 7.60]	0.178
Mayo Risk Score	−0.56 [−1.00; −0.06]	−0.48 [−1.07; 0.34]	0.565
AOS	1.52 [1.20; 1.95]	1.25 [1.06; 1.60]	0.079
LSM > 9.6 kPa	14 [26.4]	6 [14.0]	0.214
MRS > 0	12 [22.2]	13 [31.7]	0.421
ANALI score	3 [2; 3]	3 [2; 3]	0.230
ANALI score > 2	37 [68.5]	26 [60.5]	0.541
ANALI with GD ≥ 2	29 [51.8]	17 [39.5]	0.313

Abbreviations: ALP, alkaline phosphatase; ALT, alanine aminotransferase; AOS, Amsterdam Oxford Score; AST, aspartate aminotransferase; GD, gadolinium; GE, gastroesophageal varices; IBD, inflammatory bowel disease; LSM, liver stiffness measurement; MRI, Magnetic resonance imaging; MRS, Mayo Risk Score; ULN, upper limit of normal.

### 3.2 Radiomics Features Selection and Univariable Assessment in the Training Cohort

The radiomics features selected when using MRS > 0 as the outcome included *NGTDM‐Busyness* in the ADC map and *GLRLM‐Run Entropy* in FS‐T2W. Conversely, when using LSM > 9.6 kPa as the outcome, the selected features were *GLCM‐Cluster Shade* in T1W HBP, *GLDM‐Large Dependence Low Grey Level Emphasis* in the ADC map and *GLRLM‐Run Entropy* in FS‐T2W. Detailed definitions, median values and comparisons between high and low‐risk patients for these features are provided in Table S2.

The performance of the features in discriminating high and low‐risk patients at the univariate analysis is presented in Table 2.

**TABLE 2:** Radiomics features with significant association with high‐risk classification (Mayo risk score > 0 and LSM > 9.6kPa) at the univariable logistic regression analysis in the training cohort (A) and analysis of the features in the validation cohort (B).

Features	Mean odds ratio (95% CI)	Mean AUC	SD AUC	*p*
**A**				
Mayo Risk Score				
*NGTDM‐Busyness* in the ADC map	2.95 (2.24–4.79)	0.82	0.4	0.002
*GLRLM‐Run Entropy* in FS‐T2W	2.99 (2.31–4.41)	0.78	0.4	0.01
Liver stiffness measurement				
*GLCM‐Cluster Shade* in T1W HBP	4.25 (3.35–5.75)	0.85	0.3	0.001
*GLDM‐Large Dependence Low Gray Level Emphasis* in ADC	2.91 (2.32–3.84)	0.81	0.3	0.002
*GLRLM‐Run Entropy* in FS‐T2W	6.15 (4.90–8.69)	0.92	0.2	< 0.001
**B**				
Mayo Risk Score				
*NGTDM‐Busyness* in the ADC map	1.72 (0.94–3.16)	0.65	ND	0.078
*GLRLM‐Run Entropy* in FS‐T2W	3.90 (1.46–10.42)	0.84	ND	0.007
Liver stiffness measurement				
*GLCM‐Cluster Shade* in T1W HBP	0.85 (0.43–1.68)	0.59	ND	0.643
*GLDM‐Large Dependence Low Gray Level Emphasis* in ADC	1.58 (0.80–3.11)	0.71	ND	0.187
*GLRLM‐Run Entropy* in FS‐T2W	2.97 (1.33–6.66)	0.87	ND	0.008

### 3.3 Radiomics Features Assessment in the Validation Cohort

**FIGURE 1:**
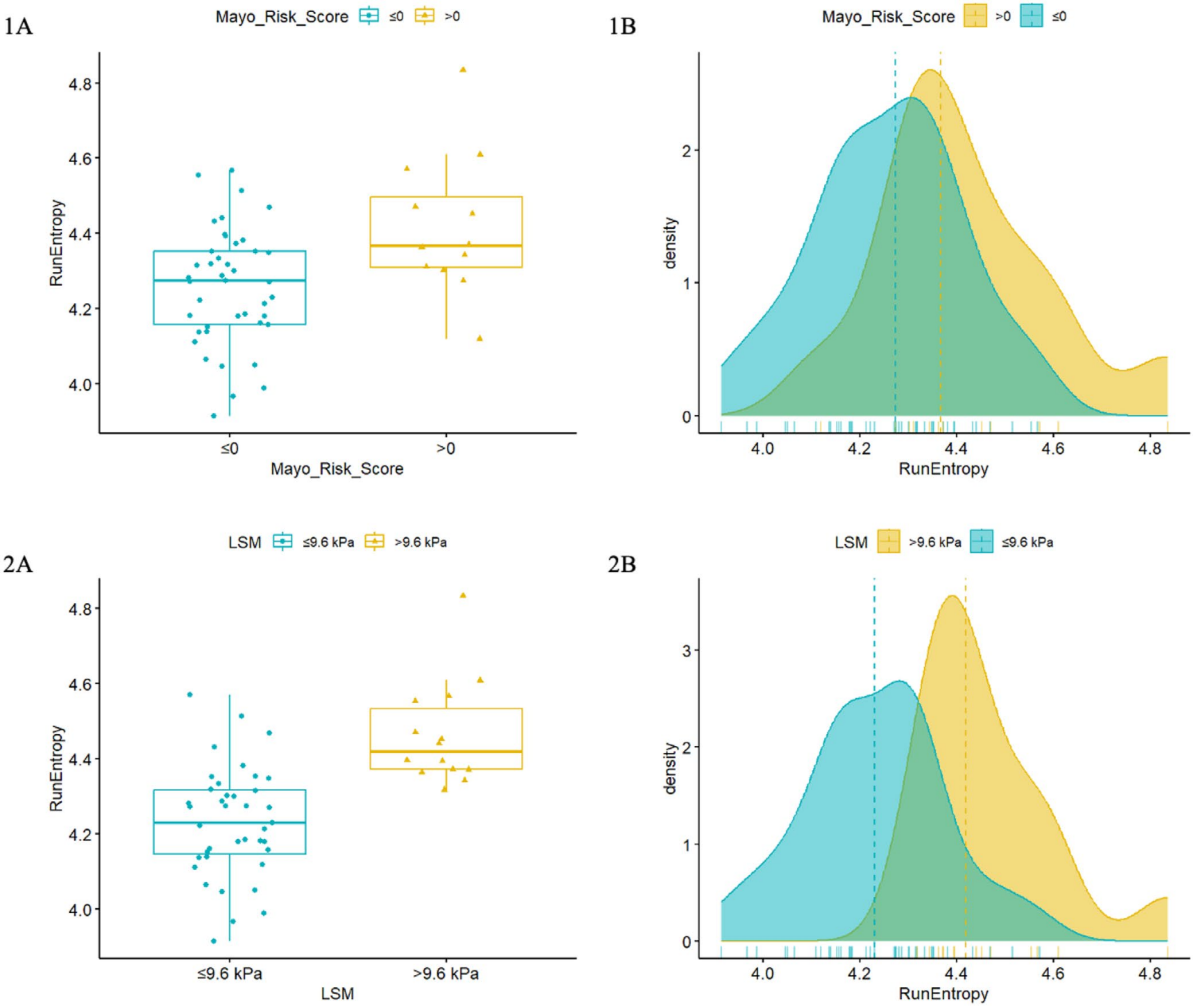
Boxplots (A) and Density plots (B) of FS‐T2W *GLRLM‐Run Entropy* in the training cohort when using MRS (A) and LSM (B) as outcome.

The radiomics features associated with high‐risk patients at the univariable analysis have been tested in the validation cohort on 41 and 39 patients using MRS and LSM as outcomes, respectively, due to missing data. Among the radiomic features identified for each of the two outcomes, only the *GLRLM‐Run Entropy* in FS‐T2W consistently demonstrated an association with patients at elevated risk of adverse outcomes (Table 2, Figure 1). When considering MRS as the outcome, this feature yielded an OR of 3.90 (CI 1.46–10.42) and an AUC of 0.84. When assessed against LSM, the OR was 2.97 (CI 1.33–6.66) with an AUC of 0.87 (Table 2).

Further details on the portability of FS‐T2W *GLRLM‐Run Entropy* over simplified liver segmentations, including the results of simplified spatial registration and region of interest definition tests, are provided in the Results, Sections 2.1: Appendix S1.

### 3.4 FS‐ *T2W* *GLRLM‐Run Entropy* for Predicting Clinical Outcomes

A time‐to‐event analysis was performed to validate the predictive ability of the selected radiomics feature towards clinical outcomes. In this analysis, 99 patients were included with a median follow‐up of 21.38 [IQR 13.83–27.93] months. Eleven clinical events (11.1%) occurred during the follow‐up period: six patients developed hepatic decompensation and five underwent liver transplantation (LT). Indications for LT were: end‐stage liver disease in four patients, hilar biliary stricture not amenable to treatment in one patient, and suspicion of hepatocellular carcinoma in advanced liver disease in one patient. The characteristics of the combined cohort are represented in Table S3.

In the univariate analysis, FS‐T2W *GLRLM‐Run Entropy* demonstrated a significant association with clinical outcomes, exhibiting a Hazard Ratio (HR) of 1.480 per 0.1 increase (95% CI 1.226–1.786, *p* < 0.001) and a C‐index of 0.857. The associations between clinical outcomes and known currently used risk stratifiers in PSC are reported in Table 3.

**TABLE 3:** Univariable and bivariable Cox models towards clinical events in the combined cohort (training + validation).

	HR (95% CI)	*p*	C‐index (95% CI)
**Univariable Cox Model**			
*FS‐T2W GLRLM‐Run Entropy*, per 0.1	1.480 (1.226; 1.786)	< 0.001	0.857 (0.783; 0.930)
Age, per 10 years	1.006 (0.970; 1.043)	0.738	0.540 (0.393; 0.687)
Sex M vs. F	3.996 (0.862; 18.522)	0.077	0.635 (0.503; 0.767)
MRS > 0 vs. ≤ 0	17.750 (3.820; 82.401)	< 0.001	0.810 (0.696; 0.923)
LSM > 9.6 kPa vs. ≤ 9.6 kPa	21.460 (4.621; 99.670)	< 0.001	0.921 (0.863; 0.979)
AOS, per unit	7.383 (2.477; 21.999)	< 0.001	0.816 (0.703; 0.928)
MELD, per unit	1.376 (1.113; 1.702)	0.003	0.727 (0.570; 0.885)
ALP × ULN, per unit	1.468 (1.264; 1.704)	< 0.001	0.769 (0.685; 0.854)
Bilirubin, per unit	1.290 (1.085; 1.535)	0.004	0.834 (0.745; 0.922)
AST × ULN, per unit	2.645 (1.832; 3.818)	< 0.001	0.871 (0.790; 0.952)
ALT × ULN, per unit	1.752 (1.349; 2.276)	< 0.001	0.807 (0.669; 0.945)
ANALI, per unit	6.770 (2.350; 19.505)	< 0.001	0.794 (0.695; 0.892)
ANALI with GD ≥ 2 vs. < 2	11.406 (1.460; 89.121)	0.020	0.716 (0.602; 0.830)
**Bivariable Cox Models**			
*FS‐T2W GLRLM‐Run Entropy*, per 0.1	1.633 (1.257; 2.122)	< 0.001	0.897 (0.831; 0.963)
AOS, per unit	8.930 (2.496; 31.949)	0.001	
*FS‐T2W GLRLM‐Run Entropy*, per 0.1	1.475 (1.210; 1.799)	< 0.001	0.885 (0.806; 0.965)
MELD, per unit	1.315 (1.060; 1.631)	0.013	
*FS‐T2W GLRLM‐Run Entropy*, per 0.1	1.241 (0.969; 1.590)	0.088	0.864 (0.792; 0.936)
ALPxULN, per unit	1.330 (1.056; 1.676)	0.015	
*FS‐T2W GLRLM‐Run Entropy*, per 0.1	1.412 (1.148; 1.737)	0.001	0.881 (0.817; 0.946)
Bilirubin, per unit	1.146 (0.925; 1.419)	0.213	
*FS‐T2W GLRLM‐Run Entropy*, per 0.1	1.283 (1.010; 1.629)	0.041	0.874 (0.808; 0.940)
ANALI, per unit	3.864 (1.227; 12.173)	0.021	
*FS‐T2W GLRLM‐Run Entropy*, per 0.1	1.350 (1.098; 1.659)	0.004	0.850 (0.752; 0.949)
ANALI GD ≥ 2 vs. < 2	5.661 (0.654; 49.012)	0.115	

Abbreviations: ALP, alkaline phosphatase; ALT, alanine aminotransferase; AOS, Amsterdam Oxford Score; AST, aspartate aminotransferase; GD, Gadolinium; LSM, liver stiffness measurement; MRS, Mayo Risk Score; ULN, upper limit of normal.

**FIGURE 2:**
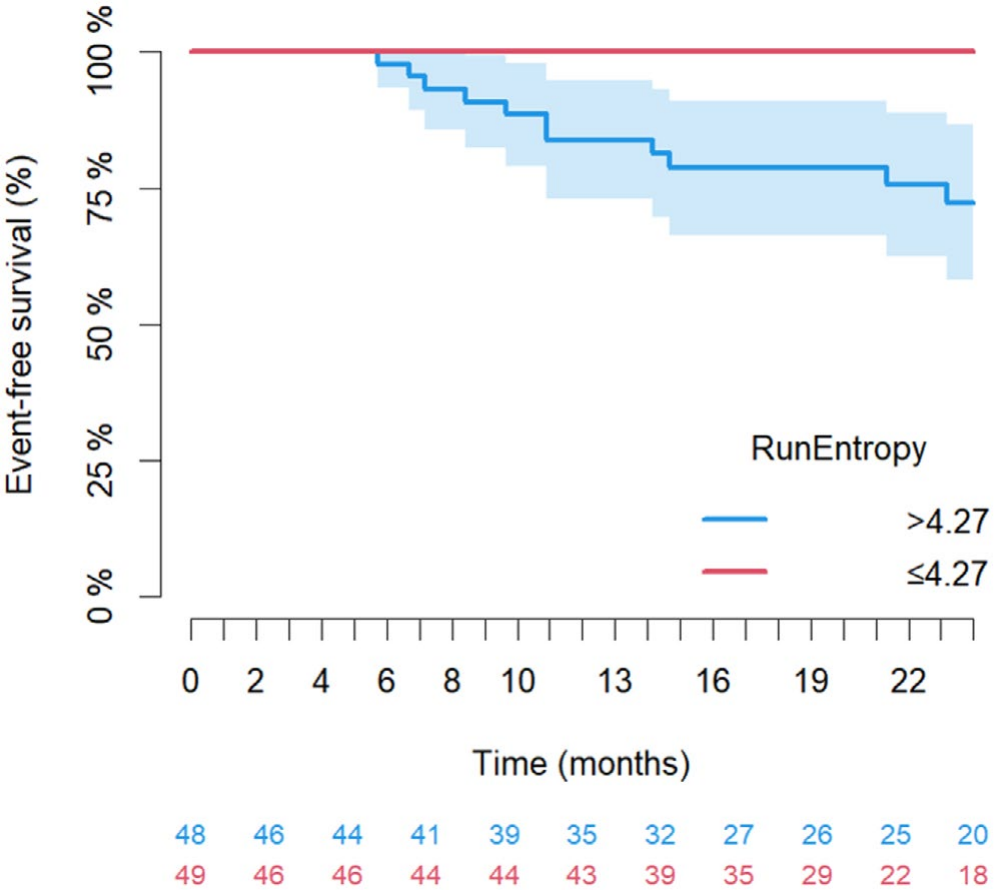
Kaplan–Meier curves for clinical outcome (liver transplantation, liver‐related death, hepatic decompensation in the combined cohort by groups according to FS‐T2W *GLRLM‐Run Entropy* split using its median value (4.27)). Log Rank < 0.0001.

To enable for a visual assessment of the discriminative power of the in FS‐T2W *GLRLM‐Run Entropy* across the combined cohort, risk groups were built using as cut off the median value of the feature in the cohort, which was 4.27. Figure 2 offers an intuitive depiction of variation in prognosis between patients according to different values of the feature (log‐rank *p* < 0.0001).

In the bivariate Cox regression models, *GLRLM‐Run Entropy* in FS‐T2W demonstrated an increase in the predictive accuracy of the known risk stratifiers, the AOS, MELD, ANALI score, ALP and bilirubin. Moreover, the feature maintained a significant and independent association with the development of clinical outcomes when analysed with AOS, ANALI score without gadolinium and MELD. Adding in FS‐T2W *GLRLM‐Run Entropy* as a covariate together with these scores significantly enhanced their predictive accuracy, increasing the C‐index for AOS from 0.816 (0.703; 0.928) to 0.897 (CI 0.831–0.963), for ANALI score without gadolinium from 0.794 (0.695; 0.892) to 0.874 (0.808; 0.940), and for MELD from 0.727 (CI 0.570; 0.885) to 0.885 (CI 0.806–0.965), as shown in Table 3.

## 4 Discussion

In this prospective, proof‐of‐concept study, we identified the radiomics feature *GLRLM‐Run Entropy*, computed in the whole liver on a standardised FS‐T2W MRI sequence, as effective in identifying PSC patients at high risk of clinical events. FS‐T2W *GLRLM‐Run Entropy* showed strong predictive performance in the validation cohort, with AUC values of 0.84 and 0.87 for MRS and LSM, respectively. Cox regression analysis on the whole cohort further confirmed its predictive power for clinical endpoints, with a HR of 1.478 per 0.1 increase and (95% CI 1.175–1.860, *p* = 0.001) and a C‐Index of 0.857.

While MRI‐MRCP is central to PSC diagnosis and management, its role in prognostic assessment remains limited due to qualitative interpretations that lead to poor reproducibility and insufficient inter‐observer agreement [1, 2]. Large variations in imaging protocols across centres also hinder the establishment of a unanimous approach for interpreting MRI‐MRCP data. Recent efforts, including our own, have explored the use of quantitative MRCP in PSC imaging analysis, focusing on biliary metrics only [5, 6, 7, 8]. However, these approaches overlook the liver parenchyma and the peri‐biliary area, limiting the breadth of the MRI application [10].

Several studies have shown that the liver atrophy and fibrosis, and the peri‐biliary inflammation are associated with the development of adverse outcomes [13, 22]. The only evidence applying quantitative image analysis (i.e., radiomics) to the whole liver in PSC patients used a topological data analysis (TDA) method based on T1W contrast‐enhanced images to predict hepatic decompensation at 1 year in a 54‐patient single‐centre cohort [9]. Despite promising results, the complexity of TDA limits its clinical applicability as it requires specialised knowledge often beyond the scope of typical clinical training, limiting its practicality in routine healthcare settings. In our study, we used PyRadiomics, an IBSI‐compliant, free and globally available software, to extract liver parenchyma radiomics features from a 3D‐ROI including the whole liver except large vessels and bile ducts.

**FIGURE 3:**
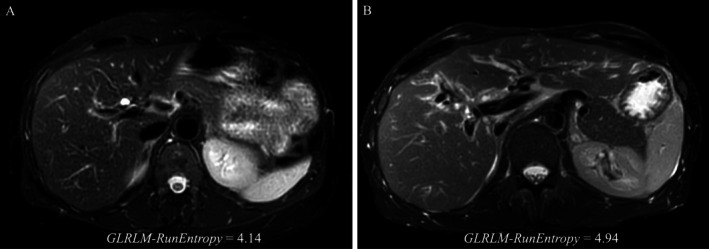
Two FS‐T2W sequences analysed in this study. Patient A is a low‐risk patient (MRS < 0, alkaline phosphatase within normal range, LSM 6.5 kPa, stable disease over time; *GLRLM‐Run Entropy* 4.14). Patient B exhibits intense fibro‐inflammatory activity (alkaline phosphatase 3.4×ULN, LSM 15.3 kPa) and underwent liver transplantation 20 months post‐MRI due to cholangiocarcinoma onset (*GLRLM‐Run Entropy* 4.94). The differences appreciable to the human eye are minimal but are quantitatively reflected in the radiomic feature.

The FS‐T2W sequence provides insights into liver parenchyma in terms of periportal edema and collagen deposition [21]. *GLRLM‐Run Entropy* measures the randomness in consecutive pixels of the same grey level intensity. Higher values of this feature in FS‐T2W sequences may reflect complex texture patterns being the quantitative expression of the inflammation and fibrosis in the peri‐biliary area of PSC patients. Figure 3 illustrates how subtle differences in texture, not easily visible to the human eye, are captured by this radiomic feature.

FS‐T2W *GLRLM‐Run Entropy* was computed within the entire liver. The computation of *GLRLM‐Run Entropy* in Pyradiomics is rapid, and the required preprocessing of FS‐T2W images is similarly time‐efficient. As detailed in the Supporting Information, precise liver segmentation is not strictly necessary; however, the ROI should cover as much of the liver parenchyma as possible to ensure feature robustness. In this study, liver segmentations were performed on T1W HBP images and subsequently spatially registered to other MR sequences. This approach was chosen for its simplicity, as T1W HBP is part of the standard protocol in our centre, and allows straightforward liver segmentations. In the absence of T1W HBP images, comparable segmentations can be obtained on portal venous phase or from non‐contrast T1W sequence images [18, 23]. Liver segmentations were carried out using ITK‐Snap for the training cohort and with an in‐house artificial intelligence (AI) algorithm for the validation cohort. Segmentation using ITK‐Snap required approximately 20 min per patient, whereas AI‐assisted segmentation in the validation set required an average of 3 min per patient for review and refinement. Although manual remains the most time‐consuming step, the increasing availability of commercial and open‐source tools for automatic liver segmentation makes broader implementation feasible.

Despite the limited number of patients enrolled in the study (i.e., in the rare disease field) potentially suggesting that the results might be compromised by overfitting, the validation of the features in an independent cohort and the performance test in a time‐to‐event analysis make the results reliable. The univariate time‐to‐event analysis data showed good predictive accuracy for the FS‐T2W *GLRLM‐Run Entropy* and for all the known PSC risk stratifiers (Table 3). To correctly power the analysis based on the cohort's size and the event number, less than three co‐variates were allowed in the Cox models. Considering the use of MRS and LMS for stratifying high‐risk patients in the selection of radiomic features, although the Cox analysis was conducted on the combined cohort with the addition of the time variable, we decided not to include them in the bivariate analysis to avoid introducing data leakage bias. Therefore, we evaluated the added value of FS‐T2W *GLRLM‐Run Entropy* in association with AOS, MELD, ALP and bilirubin. In all the models, FS‐T2W *GLRLM‐Run Entropy* increased predictive accuracy of clinical events in terms of C‐Index compared to univariate models. Furthermore, when associated with MELD and AOS, it remained independently and significantly associated with the development of clinical events (Table 3).

In our cohort, at the univariate analysis both the ANALI score with and without gadolinium were found to be predictive of clinical events (*p* < 0.001, C index 0.794 [0.695; 0.892] and *p* = 0.020, C index 0.716 [0.602; 0.830], respectively), although with lower discriminatory performance compared to FS‐T2W GLRLM‐Run Entropy (*p* < 0.0001, C Index 0.857 [0.783; 0.930]). Moreover, in the bivariate model combining FS‐T2W GLRLM‐Run Entropy with the ANALI score without gadolinium, both variables remained independently associated with clinical outcomes, and their combination resulted in improved predictive performance (C Index 0.874 [0.808; 0.940]), exceeding the predictive performance of either variable at the univariate analysis.

These findings although derived from a relatively small but prospective cohort, support the potential complementary role of quantitative radiomics and semiquantitative imaging scores in refining risk stratification. Integrating such features may enhance prognostic accuracy and reduce inter‐operator variability, offering a more robust and reproducible radiological assessment of key disease processes in PSC (i.e., fibrosis, peri‐biliary inflammation and bile duct abnormalities).

A limitation of our study is the selection of radiomic features using surrogate markers. However, there is no ground truth in PSC, that is, no established biomarkers that reliably estimate disease activity nor prognosis in PSC [1]. We have chosen a value of liver stiffness greater than 9.6 kPa, as this has been shown to be prognostic in a retrospective study [18], and preliminary data from a study under the consortium of the International PSC Study Group (FICUS study) have confirmed this cut off as prognostic in a large patient prospectively enrolled population [19]. The MRS, historically developed in tertiary and transplant centres, is widely applied as a benchmark in the development of new surrogate biomarkers for the disease [12]. Despite its good accuracy in several published cohorts [12, 23], it loses predictive accuracy beyond 4–5 years from the point of application [24]. Nonetheless, the limited follow‐up of our cohort, primarily secondary to the prospective nature of the study and the need for images acquired with a standardised protocol, renders its applicability more reliable in our context and it has an excellent predictive performance at the univariate analysis in the merged cohort. We decided not to not include ALP for feature extraction, as although it is a primary endpoint in many PSC clinical trials, it is a fluctuating marker and not always reliable in assessing disease progression in the short‐term follow‐up [25].

A second limitation of this study is the absence of external validation. We validated the predictive feature in an independent internal cohort using standardised MR sequence. The lack of robustness of radiomics features towards image acquisition protocols and scanner models is a significant challenge in quantitative medical image analysis, often resulting in reduced performance in external validations compared to internal ones. In liver MRI, slice thickness has been identified as the main factor limiting feature robustness [26], while phantom‐based data analyses have shown that both slice thickness and pixel size significantly impact reproducibility [27]. In these studies, preprocessing techniques such as voxel size resampling were found to improve feature robustness, supporting the use of standardised pipelines in multicentre settings. In line with these findings, our images were pre‐filtered, resampled onto an isotropic 3 × 3 × 3 mm^3^ voxel grid, and quantized using a 64‐bin fixed bin number (FBN) approach—precisely to standardise the main factors known to affect radiomic feature robustness in liver MRI. We hypothesise that applying the same preprocessing pipeline to similar sequences acquired on different scanners could yield comparable distributions of the selected feature. This assumption will be tested in the planned multicentre study. Should significant site effects emerge, we plan to apply harmonisation techniques such as ComBat to account for them. Moreover, we used a fat‐saturated, motion‐compensated T2‐weighted sequence that is available across different vendors and widely implemented in radiology departments (e.g., T2W‐PROPELLER on GE and T2W‐BLADE on Siemens) [26].

This first proof‐of‐concept study highlights the predictive power of a radiomics feature—FS T2W *GLRLM‐Run Entropy—*in PSC and its potential role in risk stratification.

This radiomics feature demonstrated excellent performance and was confirmed in an independent validation cohort as well as for clinical events. Its computation by means of freely available tools further supports its integration into daily practice, reinforcing FS‐T2W *GLRLM‐Run Entropy*'s potential as a quantitative, reproducible marker for liver parenchyma assessment. Its utility in conjunction with existing MRCP sequence analysis software is worth exploring in prospective studies. Such integration could offer a more comprehensive and accurate tool for evaluating liver in PSC, potentially improving patient management and outcome prediction. Further independent studies are needed to confirm this data.

## Author Contributions

Conceptualization and design of the study: L.C., E.D.B., D.P.B., M.C., C.M., D.I. Acquisition of data: L.C., E.D.B., C.M. Analysis and interpretation of data: L.C., D.P.B., M.C., D.I., C.M. Writing – original draft: L.C., E.D.B. Review and editing: all the authors. Supervision: M.C., P.I., D.I., E.D.B.

## Disclosure

Data Transparency Statement: Deidentified individual participant data that underlie the reported results will be made available 3 months after publication for a period of 5 years. Proposals for access should be sent to pietro.invernizzi@unimib.it.

## Ethics Statement

This study was approved by the University of Milan‐Bicocca Research Ethics Committee (study name: PSC Database‐Clinicaltrial.gov ID NC705618145). All participants provided written informed consent before enrollment, in accordance with the Declaration of Helsinki.

## Conflicts of Interest

The authors declare no conflicts of interest.

## Supporting information


**Data S1:** liv70348‐sup‐0001‐DataS1.docx.

## Data Availability

The data that support the findings of this study are available on request from the corresponding author. The data are not publicly available due to privacy or ethical restrictions.
