# Supplementary material for: Radiomics‐Based Prognostication in Primary Sclerosing Cholangitis: A Proof‐of‐Concept Study
Source: Liver Int. 2025 Sep 27;45(11):e70348. doi: 10.1111/liv.70348 (PMC12476022; doi:10.1111/liv.70348)
Supplement: Supplementary file 1 — Data S1: liv70348‐sup‐0001‐DataS1.docx. [file LIV-45-0-s001.docx]

### **Supplementary Materials**

### **1. Supplementary methods**

**1.1 Semiautomated liver segmentation and radiomics regions of interest (ROIs) definition**

GRE-MR images were corrected for magnetic field inhomogeneity [B4 algorithm – 3DSlicer (https://www.slicer.org/)]^1^. All the images were then filtered with the Gradient Anisotropic Diffusion filter and cropped around the liver (3D Slicer). Liver was semi-automatically segmented on T1W HBP images. On the training cohort, the ITK-SNAP (https://sourceforge.net/projects/itk-snap/) 3D active contour tool was initially used; segmentations were then manually refined by a radiologist with 15-years’ experience in abdominal MRI, particularly to check that larger vessels and larger bile duct were properly excluded. As to the validation cohort, two 3D segmentation UNets (10 layers and 1.26x10^6^ parameters the first, 8 layers and 0,60 x10^6^ parameters the second; Tensor Flow platform, https://www.tensorflow.org/), trained on the training cohort T1W HBP-segmentation pairs, were applied. Liver segmentations were computed with STAPLE (Simultaneous Truth and Performance Level Estimation) and then manually refined by a radiologist with 15-years’ experience in abdominal MRI. T1W HBP liver segmentations were then transferred to each of the other MR sequences by applying the transformation able to spatially register T1W HBP images to the relevant sequence. The ANTsPy library was used (<https://github.com/ANTsX/ANTsPy>) and the SyNRA (Rigid + Affine + deformable transformation with mutual information as optimization metric) was used to correct for any breath related mismatch. As to ADC maps, registrations were estimated on the corresponding DWI images.

**1.2 Radiomic Feature extraction and selection**

Liver radiomic features were extracted for each of the MRI sequences chosen for the analysis, both in the training and validation cohort. Radiomics features were extracted using the free and IBSI-compliant PyRadiomics tool (<https://pyradiomics.readthedocs.io/en/latest/index.html>) (parameters: isotropic voxel 2.5x2.5x2.5, quantization FBN 32 bin)^2^. Fourteen morphological features were extracted on the liver segmentation; then, for each MR sequence, 93 radiomics features were extracted including first-order statistics (18 features), gray-level co-occurrence matrix (GLCM) (24 features), gray-level run-length matrix (GLRLM) (16 features), gray-level size-zone matrix (GLSZM) (16 features), neighboring gray-tone difference matrix (NGTDM) (5 features), and gray-level dependence matrix (GLDM) (14 features). The characteristics of all the features’ classes are represented in Suppl Table 4.

**1.3 Feature selection**

Patients of the training cohort were divided into high-risk group and low-risk group. When considering MRS as outcome, the high-risk group included patients with MRS>0 and the low-risk group patients with MRS ≤0; when considering LSM as outcome, the high-risk group included patients with LSM >9.6kPa and the low-risk group patients with LSM ≤ 9.6kPa. The feature selection process described in the following was performed separately for each outcome.

Two feature selection strategies have been applied and features chosen by both strategies were finally selected to be assessed in the validation cohort.

As to the first strategy, patients have been randomly divided 100-times in 5 equally sized groups, each with a similar outcome percentage. In each of the 500 groups the association between radiomic features and the outcome have been assessed using the non-parametric Wilcoxon-Mann-Whitney test. Features with a Spearman correlation index >0.5 have been discarded and a LASSO feature selection (logistic regression model with L1 penalty, regularization parameter optimized in cross-validation) has been applied. Features selected in at least 126 groups out of 500 (>25%) have been finally kept.

As to the second strategy, a first intra-MR sequence feature selection step followed by a second inter-MR sequence feature selection step has been performed. In both steps, patients have been randomly divided 100-times in 5 equally sized groups, each with a similar outcome percentage. Clusters of highly correlated features (Spearman correlation >0.8) have been created and in each cluster the feature with the largest inter-subject variability has been selected. Features selected in at least 250 groups out of 500 (≥50%) in the first intra-MR sequence step have been given in input to the second inter-MR sequence step. Features selected in at least 250 groups out of 500 (≥50%) in the second inter-MR sequence step have been given in input to a last step in which features associated with the outcome have been selected (Wilcoxon-Mann-Whitney test with Bonferroni correction, p-value < 0.05, 500 patient subgroups created as in the previous steps; selection in at least 450 groups out of 500 (≥90%)).

**1.4 Radiomics features portability over simpler liver segmentations**

Liver segmentations have been obtained on T1W HBP images and then elastically registered to other sequences to correct for any breath-related mismatch. The ability of selected radiomic features to remain predictive if computed inside segmentations easier to obtain was assessed on the training dataset. In a first test, we simplified the spatial registration step. For MR sequences without any visual mismatch vs T1W HBP images, the liver segmentation obtained on T1W HBP images was directly used. For those showing a spatial mismatch, spatial registration was manually obtained by means of the 3D Slicer Landmark Registration tool. In the second test, radiomics features were computed for each MRI sequence into a ROI constituted by three 15 ml spheres centred on the portal vein axial plane, one the left hepatic lobe, two on the right one, properly placed to avoid major vessels or biliary ducts.

1. **Supplementary Results**

***FS-T2W GLRLM-Run Entropy* portability over simpler liver segmentations**

In the first test, we simplified the definition of the region for *GLRLM-Run Entropy* computation*,* by removing the elastic registration step to match T1W HBP liver segmentations to corresponding FS-*T2W* sequences*.* In 36/58 patients segmentation obtained on T1W HBP were directly used, while for 22/58 patients T1W HBP segmentations were spatially registered to FS-*T2W* sequences by simpler manual landmark registration. *GLRLM-Run Entropy* obtained an AUC of 0.76 and 0.91, when assessed vs MRS and LMS, respectively.

In the second test, we tried to further simplify the region definition, by replacing the whole liver segmentation with the union of three 15 ml spheres. FS-*T2W GLRLM-Run Entropy* computed on this limited region obtained an AUC of 0.46 and 0.66, when assessed vs MRS and LMS, respectively. Thus, FS T2W *GLRLM-Run Entropy* must be necessarily calculated on the entire liver parenchyma.

1. **Supplementary Tables**

Supplementary table 1

MRI scanning parameters at 1.5 T.

3D T1 FS GRE was acquired in the arterial, portal-venous and transitional phase, and repeated with a flip angle of 15 and the same parameters in the hepatobiliary phase. Sequences highlighted with (*) were used for radiomics analyses.

| **Sequence** | **Geometry** | **TR (ms)** | **TE (ms)** | **FA (α)** | **FOV (mm)** | **Th (mm)** | **Matrix (mm)** | **NSA (#)** |
| --- | --- | --- | --- | --- | --- | --- | --- | --- |
| *IP/OP T1 WI | Axial | 160 | 2.3-4.6 | 80 | 360x320 | 5 | 256x172 | 1 |
| TSE T2 WI | Coronal | 6573 | 90 | 90 | 380x380 | 5 | 360x360 | 1 |
| T2 WI - MultiVane | Axial | 2249 | 100 | 90 | 360x360 | 4 | 360x360 | 1 |
| FS T2 WI – MultiVane (T2spir) | Axial | 1809 | 80 | 90 | 350x350 | 5 | 252x252 | 1 |
| *DWI | Axial | 1895 | 65 | 90 | 360x321 | 5 | 120x105 | 1 |
| *FS T2 WI | Axial | 1000 | 320 | 90 | 350x298 | 5 | 220x162 | 1 |
| *3D T1 FS GRE | Axial | 4.0 | 1.94 | 10 | 360x320 | 5 | 256x202 | 1 |
|  | Coronal | 3.9 | 1.83 | 10 | 350x350 | 4 | 176x174 | 1 |
| IP T1 WI | Axial | 160 | 4.6 | 80 | 360x320 | 5 | 256x172 | 1 |

*TR: repetition time; TE: echo time; FA: flip angle; FOV: field-of-view; Th: thickness; NSA: number of single acquisitions; T1- or T2-WI: T1- or T2-Weighted Imaging; IP: In Phase; OP: Out-of-Phase, TSE: Turbo-Spin-Echo; FS: Fat-Suppressed, DWI: Diffusion-Weighted Imaging; GRE: Gradient Echo*

Supplementary table 2

Features’ class definitions

| Feature class | Meaning |
| --- | --- |
| Neighboring gray-tone difference matrix (NGTDM) | A Neighboring Gray Tone Difference Matrix quantifies the difference between a gray value and the average gray value of its neighbors within distance 𝛿. The sum of absolute differences for gray level 𝑖 is stored in the matrix. Let **𝐗**𝑔𝑙 be a set of segmented voxels and 𝑥𝑔𝑙(𝑗𝑥,𝑗𝑦,𝑗𝑧)∈ **𝐗**𝑔𝑙 be the gray level of a voxel at position (𝑗𝑥,𝑗𝑦,𝑗𝑧), then the average gray level of the neighborhood is:  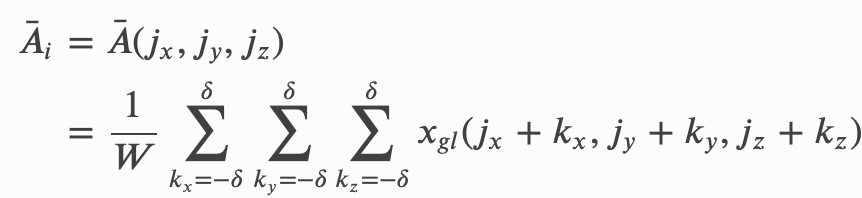  Here, 𝑊 is the number of voxels in the neighborhood that are also in **𝐗**𝑔𝑙. |
| Gray-level run-length matrix (GLRLM) | A Gray Level Run Length Matrix (GLRLM) quantifies gray level runs, which are defined as the length in number of pixels, of consecutive pixels that have the same gray level value. In a gray level run length matrix **𝐏**(𝑖,𝑗\|𝜃), the (𝑖,𝑗) element describes the number of runs with gray level 𝑖 and length 𝑗 occur in the image (ROI) along angle 𝜃. |
| Gray-level co-occurrence matrix (GLCM) | A Gray Level Co-occurrence Matrix (GLCM) of size 𝑁𝑔×𝑁𝑔 describes the second-order joint probability function of an image region constrained by the mask and is defined as **𝐏**(𝑖,𝑗\|𝛿,𝜃). The (𝑖,𝑗) element of this matrix represents the number of times the combination of levels 𝑖and 𝑗occur in two pixels in the image, that are separated by a distance of 𝛿 pixels along angle 𝜃. The distance 𝛿 from the center voxel is defined as the distance according to the infinity norm. For 𝛿=1, this results in 2 neighbors for each of 13 angles in 3D (26-connectivity) and for 𝛿=2 a 98-connectivity (49 unique angles). |
| Gray-level dependence matrix (GLDM) | A Gray Level Dependence Matrix quantifies gray level dependencies in an image. A gray level dependency is defined as the number of connected voxels within distance 𝛿 that are dependent on the center voxel. A neighboring voxel with gray level 𝑗 is considered dependent on center voxel with gray level 𝑖 if \|𝑖−𝑗\|≤𝛼. In a gray level dependence matrix **𝐏**(𝑖,𝑗) the (𝑖,𝑗) element describes the number of times a voxel with gray level 𝑖 with 𝑗 dependent voxels in its neighborhood appears in image. |

Supplementary Table 3

List of the radiomics features selected for each outcome, their meaning, their median value in low-risk and high-risk groups, and the p-value of the Wilcoxon-Man-Whitney test on the whole patient sample. Further information of the different classes of the features are reported in Supp Table 3.

| **Radiomic features** | | **Meaning** | **Median values**  **Low-Risk vs High Risk** | **p value** | |
| --- | --- | --- | --- | --- | --- |
| **Mayo risk score** | | | | |  |
| *NGTDM-Busyness* in the ADC map | A measure of the change from a pixel to its neighbor. A high value for busyness indicates a ‘busy’ image, with rapid changes of intensity between pixels and its neighborhood. | | 14.03/18.07 | 0.004 | |
| *GLRLM-Run Entropy* in FS-T2W | RE measures the uncertainty/randomness in the distribution of run lengths and gray levels. A higher value indicates more heterogeneity in the texture patterns. | | 4.24/4.38 | 0.005 | |
| **LSM** | | | |  |  |
| *GLCM-Cluster Shade* in T1W HBP | | It is a measure of the skewness and uniformity of the GLCM. A higher cluster shade implies greater asymmetry about the mean. | -171.06/-35.18 | 0.000 | |
| *GLCM-Maximal Correlation Coefficient* in T1W arterial phase | | The Maximal Correlation Coefficient is a measure of complexity of the texture. | 0.61/ 0.74 | 0.001 | |
| *GLDM-Large Dependence Low Gray Level Emphasis* in ADC map | | Measures the joint distribution of large dependence with lower gray-level values. | 0.47-0.73 | 0.001 | |
| *GLRLM-Run Entropy* in FS-T2W | | Measures the uncertainty/randomness in the distribution of run lengths and gray levels. A higher value indicates more heterogeneity in the texture patterns. | 4.23-4.39 | 0.000 | |

Supplementary Table 4 Merged (training + validation) cohort patients’ characteristics (n=99).

| **Demographics & clinical variables** | **Median or N[Q1-Q3 or %]** |
| --- | --- |
| Age at MRI (years) | 41.59 [28.00, 56.00] |
| Female gender | 43 (44.3) |
| IBD (%) | 67 (67.68) |
| MRS (median [IQR]) | -0.52 [-1.02, 0.03] |
| LSM (kPa) | 6.10 [4.82, 8.80] |
| MELD (median [IQR]) | 7.00 [7.00, 9.00] |
| AOS (median [IQR]) | 1.37 [1.14, 1.87] |
| Cirrhosis (%) | 9 (9.3) |
| Previous.GI.bleeding | 2 (2.1) |
| ALPxULN | 1.03 [0.67, 2.09] |
| Total bilirubin (mg/dl) | 0.67 [0.50, 1.30] |
| ASTxULN (median [IQR]) | 0.78 [0.53, 1.27] |
| ALTxULN (median [IQR]) | 0.83 [0.50, 1.42] |
| MRS>0 (%) | 25 (26.3) |
| LSM>9.6 kPa | 20 (20.8) |
| Follow-up time, months (median [IQR]) | 21.4 [13.8, 27.9] |
| Clinical endpoints  LT  Liver-related death  Hepatic decompensation | 11 (11.1)  5 (5.1)  0 (0)  6 (6.1) |
| *GLRLM-Run Entropy* in FS-T2W | 4.26 [4.16, 4.36] |

*Abbreviations*: IBD: inflammatory bowel disease; GE: gastroesophageal varices; ALP: alkaline phosphatase; AST: aspartate aminotransferase; ALT: alanine aminotransferase; ULN: upper limit of normal; LSM: liver stiffness measurement; MRS: Mayo Risk Score., AOS: Amsterdam Oxford

**References**

1. Fedorov A, Beichel R, Kalpathy-Cramer J, et al. 3D Slicer as an image computing platform for the Quantitative Imaging Network. *Magn Reson Imaging*. 2012;30(9):1323-1341. doi:10.1016/J.MRI.2012.05.001

2. Van Griethuysen JJM, Fedorov A, Parmar C, et al. Computational radiomics system to decode the radiographic phenotype. *Cancer Res*. 2017;77(21):e104-e107. doi:10.1158/0008-5472.CAN-17-0339/SUPPLEMENTARY-VIDEO-S2
